# Six ways to handle dependent effect sizes in meta-analytic structural equation modeling: Is there a gold standard?

**DOI:** 10.1017/rsm.2024.10

**Published:** 2025-03-13

**Authors:** Zeynep Şiir Bilici, Wim Van den Noortgate, Suzanne Jak

**Affiliations:** 1 University of Amsterdam, Amsterdam, The Netherlands; 2 KU Leuven, Leuven, Belgium; 3 imec-ITEC, Leuven, Belgium

**Keywords:** dependent effect sizes, MASEM, meta-analysis, structural equation modeling, univariate three-level modeling

## Abstract

The current meta-analytic structural equation modeling (MASEM) techniques cannot properly deal with cases where there are multiple effect sizes available for the same relationship from the same study. Existing applications either treat these effect sizes as independent, randomly select one effect size amongst many, or create an average effect size. None of these approaches deal with the inherent dependency in effect sizes, and either leads to biased estimates or loss of information and power. An alternative technique is to use univariate three-level modeling in the two-stage approach to model these dependencies. These different strategies for dealing with dependent effect sizes in the context of MASEM have not been previously compared in a simulation study. This study aims to compare the performance of these strategies across different conditions; varying the number of studies, the number of dependent effect sizes within studies, the correlation between the dependent effect sizes, the magnitude of the path coefficient, and the between-studies variance. We examine the relative bias in parameter estimates and standard errors, coverage proportions of confidence intervals, as well as mean standard error and power as measures of efficiency. The results suggest that there is not one method that performs well across all these criteria, pointing to the need for better methods.

## Highlights

- **What is already known?**

Dependent effect sizes are very common in research synthesis; researchers may measure the same variables using multiple samples, operationalizations, or across multiple time points, leading to multiple effect size measures for the same relationship of interest coming from the same study. In a similar manner, we can come across dependent effect sizes also in the context of meta-analytic structural equation modeling (MASEM).

- **What is new?**

Applied researchers have been using several different methods in situations where dependent effect sizes are present; however, the performance of these methods in the context of MASEM had not been assessed before.

- **Potential impact for RSM readers**

We found that not one method performed well across all evaluation criteria. We also found that the method that was seen as the “gold standard” did not perform well, which is quite crucial for both applied and theoretical researchers to be aware of.

## 1 Six ways to handle dependent effect sizes in MASEM: Is there a gold standard?

Meta-analytic structural equation modeling (MASEM) is a technique that has gained in popularity in recent years as it allows researchers to meta-analyze the relationships between multiple variables in a given SEM model.^1^^–^ ^3^ Whereas traditional meta-analytic methods analyze each bivariate relationship separately, MASEM allows researchers to meta-analyze all relationships simultaneously and evaluate complete SEM models.^4^^,^ ^5^ One of the widely used MASEM techniques is two-stage SEM (TSSEM), which is the approach we focus on in the current paper. In the first stage, an overall correlation matrix is estimated based on the observed correlation matrices from individual studies using multivariate meta-analysis. In Stage 2, the SEM model is fitted on this synthesized matrix to estimate the path coefficients using weighted least squares (WLS) estimation. When pooling the correlation coefficients together in Stage 1, researchers can choose between fixed-effects and random-effects models, depending on their assumptions regarding the studies included in the analysis.^6^ Given its flexibility, the random-effects model is used more frequently and can be specified as follows:(1)

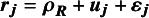

where 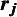 is the vector of correlations from study j, 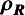 is the means of the population correlation coefficients across studies, 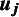 is a vector containing the deviations of the population correlation coefficients in study j from 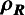 , and 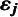 is the vector of sampling deviations of study j. The between-studies covariance matrix, **T**^**2**^, contains the covariances of 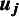 ; 
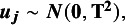
 and the within-studies covariance matrix of study j, **V**_**j**_, is the covariance matrix of the sampling deviations; 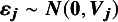 . Stage 1 results in an estimated pooled correlation matrix and its asymptotic sampling covariance matrix.^6^ In Stage 2, the pooled correlation matrix is used as the observed correlation matrix and the asymptotic sampling covariance matrix **A** is used as the weight matrix in the WLS estimation, minimizing the fit function^7^:(2)

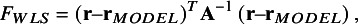



where **r** is a column vector of the unique elements in the pooled correlation matrix, **r**_MODEL_ is a column vector of the unique elements in the model implied correlation matrix, **A** is the asymptotic covariance matrix of the pooled correlations from Stage 1. For a more detailed description of TSSEM, readers can refer to Cheung,^8^ Cheung and Chan,^6^ and Cheung.^9^

**Figure 1:**
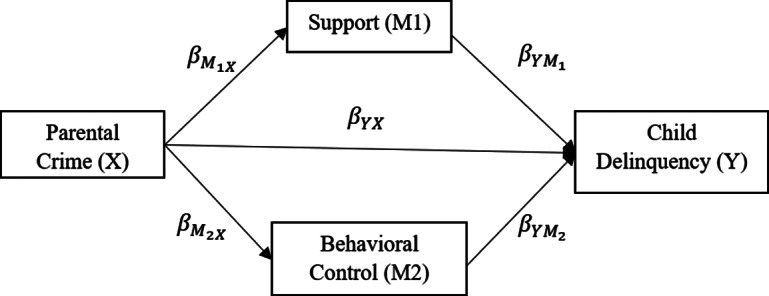
*Partial mediation model from empirical example by Stolwijk et al*.^10^

Let us now focus on an empirical application of MASEM, namely the meta-analysis of intergenerational continuity of criminal behavior where the authors specified a partial mediation model as in Figure 1.^10^ The authors hypothesized that parental support and behavioral control act as mediators for the relationship between parental crime and child delinquency. To evaluate this proposed path model on meta-analytic data, the authors gathered individual correlation matrices from 140 studies. For studies that included all variables, the correlation matrices were of dimensions four by four, containing six informative correlation coefficients. For studies that do not include all variables of interest, there were missing values in the correlation matrices. An advantage of MASEM is that the studies of interest do not need to measure every variable of interest to be included in Stage 1.^11^ This way, the analysis can take into account any study that includes at least two of the variables of interest and makes the most of the available information in existing literature.

## 2 Dependency between effect sizes

In traditional meta-analysis, dependent effect sizes arise when a study reports multiple effect size values. For example, there could be multiple informants, multiple time points, or multiple operationalization strategies.^12^^,^ ^13^ Another scenario which could lead to dependent effect sizes is when separate studies are being conducted by the same researchers, or in the same laboratory.^14^ The first scenario is usually referred to as the correlated effects structure, and the second scenario as the hierarchical effects structure.^14^ The dependence structure of interest for the current paper more closely resembles the correlated effects structure. Reviews of standard meta-analyses suggest that a large percentage of the included studies contain such dependent effect sizes,^15^ and an average of 3.6 effects per study is reported.^16^ The same scenarios also take place in the context of MASEM, where a study may provide multiple effect sizes for the same bivariate relationship (the same cell in a study’s correlation matrix). In the context of the Stolwijk et al.^10^ example, a study might assess the relationship between parental crime and delinquency among children across multiple time points or operationalize delinquency using two different questionnaires. Stolwijk et al.^10^ reported that 72.1% of the studies in their analysis included dependent effect sizes, where there were multiple values reported for the same cell in a study’s correlation matrix. To clarify the structure of a dataset in such cases, Table A1 in the Appendix provides a hypothetical dataset with two studies that report multiple effect sizes for some of the six relationships in our example.

Currently, there are no guidelines on how dependent effect sizes should be handled in the context of MASEM. Sheng et al.^11^ reviewed applications of MASEM and found that 22.7% of the studies with dependent effect sizes treated these as independent and/or used an average within the study, while 77.3% picked one effect size and disregarded the rest. Wilson, Polanin, and Lipsey^17^ proposed an alternative approach using three-level modeling at Stage 1 of MASEM to take dependent correlations into account. Their approach is being applied in practice,^18^^,^ ^19^ and referred to as the gold standard,^20^ although its statistical performance has never been evaluated. The aim of our study is therefore to evaluate how different methods of dealing with dependent effect sizes^i^ in MASEM affect the obtained results. In the next sections, we provide more detail on the approaches under evaluation.

## 3 Current strategies

The current strategies for dealing with dependent effect sizes in standard meta-analyses mainly show variation in terms of whether they remove or explicitly model the dependency.^12^ The following section details the most common methods used to deal with dependent effect sizes in the context of traditional meta-analysis. What is important to note here is that while these methods have been compared in terms of their performance in the context of traditional meta-analysis where researchers are interested in only one relationship, there has been no comparison of their performance within the context of MASEM. Our study is the first simulation study evaluating these methods of dealing with dependent effect sizes in the context of MASEM.

### 3.1 Aggregation

With the aggregation approach, the dependent effect sizes within each study are averaged so that there is only one effect size per study. Simple aggregation involves calculating the mean of the dependent effect sizes and using that value as the observed effect size for that study.^9^^,^ ^21^ Weighted aggregation, on the other hand, involves the use of weights in averaging the effect sizes. The inverse of the sampling variances can be used as weights, which will make sure that more precise estimates get more weight and have more influence on the final results.^21^ The sampling variances were calculated using the formula below:(3)

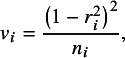



where 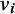 is the sampling variance for correlation 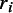 , and 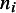 is the sample size for that correlation coefficient.^22^ Using the inverse sampling variance as weights is equivalent to conducting a fixed-effects meta-analysis within studies.^23^ An alternative approach would be to also take into account the variability of the effects in the population, however, we chose to follow the same approach as in the study by Stolwijk et al.,^10^ and create an opportunity to evaluate their conclusions with a simulation study.

The main drawback with aggregation is that there is substantial information loss, which has serious implications on statistical power.^9^ Also, the correct standard error of the average effect depends on the correlations between the effect sizes, which are seldom known.^12^^,^ ^ii^ In addition, aggregation limits the amount and extent of research questions one could ask with regard to the sources of heterogeneity in effect sizes since variance between effect sizes within studies is removed.^13^^,^ ^24^ Both the simple and weighted aggregation techniques suffer from these drawbacks, but the weighted aggregation may have a slight edge given that it does not assume that the sample sizes are comparable and that the population effect sizes are equal.^10^

### 3.2 Elimination

The elimination strategy involves selecting one effect size per study and could be done through random selection or based on a priori selection rules. For example, researchers may choose the effect size that has the highest value, or choose the effect size estimate coming from the measure with the highest reliability in the presence of multiple questionnaires to measure the same variable.^11^ With elimination, dependency among the effect sizes within the same study is eliminated, but there is information loss, which results in loss of precision,^11^^,^ ^12^ and when the effect size selection is done based on the size of the effect, this will naturally introduce bias. An assumption behind the elimination approach is that the dependent effect sizes within a study are equivalent, while in fact the result of the meta-analysis can show differences based on which effect size was selected.^12^ Much like the aggregation approach, the loss of information also affects statistical power, the precision of estimates, and the ability to evaluate heterogeneity between effect sizes within studies.^9^

### 3.3 Ignoring dependency

The ignoring dependency approach involves treating every reported effect size in a study as if they are coming from independent studies. This approach ignores the overlap in the information that each effect size provides.^24^^,^ ^25^ The uncertainty in effect sizes is thus underestimated, which leads to smaller standard error estimates, narrower confidence intervals, and increased Type I error rates in traditional meta-analysis.^21^^,^ ^25^ Ignoring dependency also means that studies that report more effect sizes will have a considerably greater influence on the results of the analysis compared to studies that report only one effect size.^13^

The three approaches outlined above can easily be implemented in the context of MASEM. For the aggregation method, the multiple effect sizes for each bivariate correlation are averaged so that there is only one effect size per each bivariate relationship in each study. In the elimination approach, one effect size is selected amongst many for each bivariate relationship with multiple reported values. The implementation of the ignoring dependency approach to the context of MASEM follows the logic of treating the multiple effects as if they are coming from different studies by treating each row in Table A1 as an independent study.

### 3.4 Wilson–Polanin–Lipsey (WPL) approach

In contrast to the approaches detailed above, the WPL approach has been specifically designed to be used in the context of MASEM. The WPL approach merges the ideas of three-level meta-analysis with MASEM by modeling the hierarchical structure in the data whereby participants are nested within effect sizes, which are nested in studies.^17^ This approach uses a multilevel mixed-effects meta-regression model with no intercept to estimate the pooled correlations at Stage 1 of MASEM:(4)

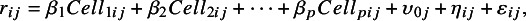



where 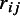 is an observed correlation *i* from study *j*, the *Cell* variables are dummy variables for each of the *p* cells in the correlation matrix, 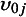 is the Level 3 (study level) random effect; 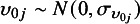 , 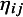 is the Level 2 (effect size level) random effect; 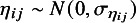 , and 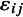 is the sampling error for correlation *i;* 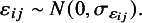 Because the intercept is excluded from the model, the 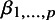 terms can be interpreted as the synthesized correlation coefficients for the *p* cells. Wilson et al.^17^ proposed using sample sizes as weights at Stage 1. A remarkable feature of this model is that the two random effects ( 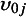 and 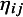 ) and the error term ( 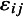 ) are specified in such a way that their variance components ( 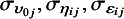 ) are assumed to be equal across cells. These terms represent the between-studies variance, within-study variance, and sampling variance, respectively. It is unclear what the effects are of making this assumption when it is not met.

Stage 2 of the WPL approach resembles Stage 2 of TSSEM; using the inversed asymptotic covariance matrix of the pooled correlations obtained in Stage 1 as the weight matrix for fitting the SEM model using weighted least squares estimation. A possible advantage of the WPL approach is that all the available information is used in the analysis, which may lead to more statistical power than the aggregation and elimination approaches and keeps the possibility of examining heterogeneity between effect sizes. Stolwijk et al.^10^ compared elimination, aggregation, and ignoring dependency approaches along with the WPL approach in an empirical application of MASEM and found that different methods lead to different results, so much so that the implications in terms of statistical significance are different. The authors note that whilst the aggregation and random selection approaches seem to underestimate the precision of the estimates, the ignoring dependency approach seems to overestimate it,^10^ which is in line with the results from the context of traditional meta-analyses.^13^^,^ ^25^ They conclude by advising the use of the WPL approach, as it is the only one modeling the dependency between effect sizes.^10^

Our study serves as the first simulation study evaluating the WPL approach, and the first study comparing the results of the discussed methods for dealing with dependent effect in the context of MASEM. The following section describes the details of the simulation study.

## 4 Simulation study

### 4.1 Data generation

**Figure 2:**
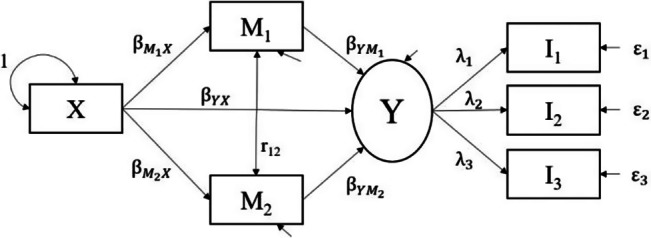
Population model for the three indicator setup.

To evaluate the performance of the different approaches outlined above, meta-analytic datasets were generated across various conditions. For the data generation mechanism, a partial mediation model was set with one independent variable, two mediator variables, and one dependent variable based on the model used in the study by Stolwijk et al.,^10^ depicted in Figure 1. For creating the dependent effect sizes, we generated the data from a model in which the outcome variable was set as a latent variable with multiple indicators, as shown in Figure 2. This closely resembles the scenario where the same construct is measured using different instruments, which has been identified as a common source of dependency in effect sizes.^26^ These multiple effect sizes can also be thought to be stemming from the existence of multiple measurement points or informants, which shows that the setup is also applicable to other sources of dependency.

For the purposes of the current paper, we set the population models to have either three or nine indicators. We assume that the different studies then use the same indicators or a subset of them, meaning that the indicators are treated as fixed. Based on the population model with three indicators for the latent variable (see Figure 2), a model-implied correlation matrix among the six observed variables was calculated. This correlation matrix served as the average population correlation matrix. We set the between-studies covariances to zero, and the between-studies variances of the correlations (**T**^2^) to various values depending on the condition. Next, for generating one meta-analytic dataset with *j* studies, *j* population correlation matrices were drawn based on the average population correlation matrix and **T**^2^, followed by drawing one sample correlation matrix per study, using the specific functions in the metaSEM package.^4^ These sample correlation matrices serve as the individual correlation matrices extracted from each study. Having three indicators for Y leads to correlation matrices of dimensions six by six (see Table 1), while the model that we will fit to the data contains four variables, that is, M1, M2, X, and Y. The existence of multiple indicators for variable Y leads to multiple correlations between indicators and the other three variables in the model. These correlations of the three indicators with the X, M1 and M2 variables thus serve as the multiple versions of the correlation between outcome variable Y with variables X, M1 and M2. In the way we generate data, if we consider all the indicator variables as separate variables, there is a maximum of one correlation per cell of the 6 × 6 matrix. However, we consider the three indicator variables as the indicators of one single variable. This merging creates multiple correlations for some cells of a 4 × 4 matrix and these multiple correlations then act as our dependent effect sizes. Table 2 provides an example of the structure of a generated dataset.

Reviews of traditional meta-analyses showed that dependent effect sizes generally occur in 57–70% of the studies included in a meta-analysis.^15^^,^ ^16^^,^ ^27^ More specifically, in the context of MASEM, Stolwijk et al.^10^ report that dependent effect sizes were reported in 72% of the studies. We therefore applied a random selection procedure such that 70% of the studies contain dependent effect sizes. For the condition where we had three dependent effect sizes, information was deleted such that 30% of studies reported only one effect size per bivariate relationship, 35% reported two effect sizes, and 35% reported three effect sizes. In conditions with nine dependent effect sizes, 10% of studies report either two or three effect sizes per bivariate relationship, 10% report four effect sizes, and so on to reach a total of 70% studies reporting multiple effect sizes for the same bivariate relationship. For the correlations concerning the other cells (such as X_M1 in Table 2), we implemented a random deletion procedure across studies to reflect the real-life situation of not every single study reporting the full correlation matrix, whereby 15% of the values reported in the columns not concerning the dependent variable were deleted. This number was chosen arbitrarily, and can easily be modified in future studies. As mentioned previously, for studies with dependent effect sizes, the additional values referring to the correlations between the variables M1, M2, and X were replaced with NA values. This way the individual studies only include extra correlations for the relationships between variable Y and the other variables, which then act as the dependent effect sizes, resulting in a structure as in Table 2. The associated R code with the procedures detailed above is provided in the OSF project page.^iii^ The following section details the different conditions of the simulation study.

**Table 1:** Example average correlation matrix for the three-indicator setup where indicators have equal loadings of 0.70

	M1	M2	X	I1	I2	I3
M1	1.000					
M2	0.240	1.0000				
X	0.200	0.2000	1.000			
I1	0.188	0.188	0.126	1.000		
I2	0.188	0.188	0.126	0.420	1.000	
I3	0.188	0.188	0.126	0.420	0.420	1.000

**Table 2:** An illustration of the data structure with dependent effect sizes

Study	X_M1	X_M2	X_Y	M1_M2	M1_Y	M2_Y
1	0.200	0.200	0.126	0.240	0.188	0.188
			0.126		0.188	0.188
			0.126		0.188	0.188

*Note:* For illustration purposes, we have re-used the values from Table 1, and have thus ignored sampling error.

## 5 Manipulated factors

### 5.1 Varying the number of dependent effect sizes per study

The number of indicators of the latent Y variable specified in the population model (see Figure 2) determines the maximum number of dependent effect sizes we can have for one bivariate relationship in the same study. For the current study, we evaluated conditions with three or nine indicators in the data-generating model. The population model for the three indicator setup is set to include six variables; M1, M2, X, I1, I2, and I3, with the last three as indicators of our latent Y variable, which makes the population correlation matrix a 6 × 6 matrix as seen in Table 1. In the nine-indicator conditions, there are instead 12 variables in total, so the initially generated correlation matrices have dimensions 12 by 12.

### 5.2 Varying the size of dependency and the exchangeability of the effect sizes

In our data generation setup, the size of the factor loadings (λ) reflects how much information the single indicators share with the latent Y variable and these values could be the same for all or vary. The size of the loadings also indirectly determines the correlation between the dependent effect sizes. To manipulate the size of the dependency between the effect sizes, we used two mean values for λ: [0.70, 0.30]. For a λ value of 0.70, the correlation between the multiple effect sizes comes out as 0.475, and for a λ value of 0.30 as a correlation of 0.086.^iv^ These values are in accordance with the values used in the simulation study by Van den Noortgate et al.^13^ The correlations between the dependent effect sizes have been shown to influence the performance of the methods in the context of traditional meta-analysis,^25^ which is why it is also important to assess their influence in the context of MASEM. In all conditions, the residual variances were chosen such that the total variances associated with each indicator were equal to 1. This was done so that the resulting matrices acted as correlation matrices and could directly be included in the MASEM analysis. Moving forward we will refer to the conditions where λ = 0.30 as small dependency between dependent effect sizes, and where λ = 0.70 as large dependency.

When the factor loadings, as specified by λ_1,_ λ_2,_ and λ_3_ values in Figure 2, are equal, the effect sizes can be considered parallel. Having parallel effects means that they are interchangeable and that they provide the researchers with the same amount of information. In the current study, this interchangeability is manipulated by varying the pattern of the factor loadings. We created three levels to correspond to different levels of interchangeability: [λ, λ, λ], [λ − 0.10, λ, λ + 0.10], and [λ − 0.20, λ, λ + 0.20]. So, in conditions with λ = 0.30, the last condition would have factor loadings of [0.10, 0.30, 0.50]. For the conditions with nine dependent effects, the pattern specified was repeated three times to fit the number of effect sizes. Changing the pattern of loadings also had implications on the size of dependency between the effect sizes; for λ = 0.30 the correlations between the multiple effect sizes ranged between [0.058, 0.115] when the loading pattern had differences of 0.10, and between [0.029, 0.144] when the loading pattern had differences of 0.20. For λ = 0.70 the correlations between the multiple effect sizes ranged between [0.405, 0.544] when the loading pattern had differences of 0.10, and between [0.337, 0.615] when the loading pattern had differences of 0.20. In the following text, we refer to the different levels of interchangeability as follows: [λ, λ, λ] as exchangeable, [λ − 0.10, λ, λ + 0.10] as small disparity, and [λ − 0.20, λ, λ + 0.20] as large disparity.

Whether the dependent effects are parallel or not has implications on the performance of the methods outlined above in terms of dealing with dependent effect sizes. If the effect sizes are truly parallel and interchangeable, then the elimination and aggregation strategies are expected to be not so harmful for parameter estimation as the assumption of homogeneous effect sizes holds. In the case of elimination, it does not matter what effect size is selected among the many since they all provide the same amount of information. Furthermore, for the random elimination and aggregation strategies, even when the effect sizes are not interchangeable, the parameter estimates should still be not very biased given that the mean value still approximates the population value, and effect sizes are averaged or eliminated randomly. On the other hand, selecting only the largest effect size is expected to lead to biased parameter estimates.

### 5.3 Varying the number of studies

To be able to generalize the findings of this simulation study, we also need to take into account that in meta-analyses, the number of studies and the sample sizes of these studies also show variation. In their review article, Fernández-Castilla et al.^26^ detail the summaries of meta-analytic studies across different fields of research. In the context of social and behavioral sciences, the article reports a wide range of the number of primary studies included in the meta-analysis, from 5 to 456 with a mean of 64.7. To reflect this in our simulation study, we varied the number of studies to take the following values: [20, 60, 100]. For the sample sizes of the primary studies, we opted to randomly sample from a lognormal distribution, i.e. 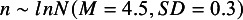 ,^v^ to determine the sample sizes within studies as in Van den Noortgate et al.^25^ and rounded them to integer values when necessary.

### 5.4 Varying the between-studies variance

The between-studies variance determines the extent to which the different bivariate relationships vary across studies. This was an especially relevant factor to manipulate given how in the WPL approach, the variances of the random effects are assumed to be equal across correlation coefficients. We evaluated two conditions, both with diagonal matrices, which meant the between-studies covariances were all set to 0. In one condition, the variances for all population correlations were set to be 0.01, whereas, in the other condition, the variances for the population correlations corresponding to the dependent effect sizes (involving variable Y) were varied using population values of either 0.01 or 0.03 (see Table A2 in the Appendix). We have chosen to use the values of 0.01 and 0.03, in accordance with the findings from the review article by Fernández-Castilla et al.^26^

### 5.5 Varying the coefficient of the causal path between X and Y

The population path coefficient of variable X to variable Y ( 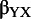 ) was manipulated to take one of two values: [0.0, 0.2]. The case when this path coefficient is kept at 0 provides us with an opportunity to calculate the false positive rate, that is, the Type I error rate, associated with that estimation. The other path coefficients in the population model are kept the same across the conditions for ease of interpretation: 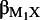 , 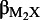 , 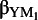 , and 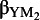 values, as well as the correlation between the two mediators, are fixed at 0.20.

In total, we analyzed 2 × (number of dependent effect sizes) × 2 (size of dependency) × 3 (exchangeability of effect sizes) × 3 (number of studies) × 2 (between-studies variance) × 2 (coefficient of the path between X and Y) = 144 conditions, and for each condition, we generated 1,000 meta-analytic datasets. We fit the model from Figure 1 to each of the datasets using six different strategies for handling the dependent effect sizes; applying TSSEM using simple averaging, weighted averaging, random selection, selecting the largest effect size, ignoring dependency, and the WPL approach with sample size weighting.

All analyses were conducted using R (version 4.1.2)^28^ and with the metaSEM package (version 1.3.0).^4^ Additionally, for the WPL method, we made use of the metafor package (version 4.2.0).^29^ Additional packages like dplyr (version 1.0.7),^30^ tidyr (version 1.1.4),^31^ and corpcor (version 1.6.10)^32^ were used when necessary. In plotting the graphs, we also made use of the ggplot2 package (version 3.3.5)^33^ and the jtools package (version 2.2.0).^34^ Interested readers can refer back to the OSF page to look at the code used in the paper as well as example code for each of the approaches using one generated dataset. The performance of these six strategies was assessed by means of multiple criteria, the details of which are presented in the following section.

### 5.6 Evaluation criteria

#### 5.6.1 Convergence

Since Stolwijk et al.^10^ reported that there were convergence issues for the random selection and averaging effect sizes strategies, we decided to further explore this in our study. We assessed the convergence of the methods by checking the status provided by OpenMx^35^ at both stages of the MASEM procedure for each replication. The metaSEM output provides us with the OpenMx statuses and how to interpret them; for OpenMx statuses of 0 and 1 the estimation is considered to have run with no problems, and any other number points to estimation problems as stated in the output. Thus, we enlisted a check whereby we first identify the problematic iterations based on their OpenMx status at Stage 1 of TSSEM. In case of non-convergence, we applied the rerun() function from the metaSEM package once which tried 10 times to find a solution, in line with the suggested practice.^36^ The replications that still did not converge were left as non-converged and thus their Stage 2 results could not be obtained. We also inspected the OpenMx status of the available replications at Stage 2 and inspected for the existence of any patterns across the different conditions.

#### 5.6.2 Bias

The parameter estimates of the different path coefficients, which are the meta-analytic effect sizes of interest in the current study, and their associated standard errors were assessed in terms of their relative bias. We focused on the path coefficients involving the variable Y for which there were dependent effect sizes present, that is, 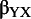 , 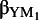 , 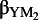 . The relative percentage bias in parameter estimates was assessed via the following formula:(5)

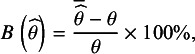

 where 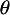 is the population value of the specific path coefficient, and 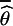 is the mean of the parameter estimate across 1,000 replications. We used a cutoff of 5% as our evaluation criterion.^37^ In order to evaluate bias in the standard errors we used the following formula:(6)

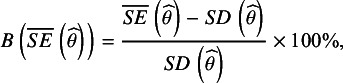



where 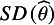 is the standard deviation of the parameter estimates across the 1,000 replications, and 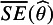 is the mean of the standard errors of the parameter estimates across the replications. We considered standard error bias less than 10% as acceptable, in accordance with previous literature.^37^ Additionally, upon request from reviewers, we assessed the average absolute bias in both parameter estimates and standard errors. It is important to also assess the absolute bias since a relative bias of 5% may have different practical significance for different population values. The absolute bias measures are directly related to the relative bias measures, and they only differ in terms of whether there is a value in the denominator or not. While the relative bias measures divide the deviation of estimates from the population value by the population value, the absolute bias measures do not and thus provide us with the raw bias values.

#### 5.6.3 Efficiency

By looking at the standard deviation values of the parameter estimates across replications 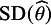 , in addition to bias calculations, we were able to comment on whether the different methods show variation in terms of efficiency. The smaller 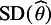 , the more efficient the estimation method is, as smaller values indicate that the parameter estimates are consistent across different iterations.

#### 5.6.4 Root mean square error

We also assessed how well the methods perform in estimating the parameters by looking at the root mean square error (RMSE) values. RMSE is a measure of the average deviation of an estimate from the population value for that parameter,^38^ and points to better prediction with lower values. RMSE can be formulated to reflect how it is a combination of bias in and variance of parameter estimates^39^:(7)

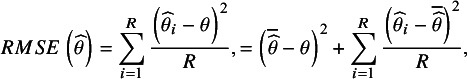

where 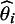 is the estimate of the population parameter 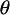 for the 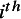 replication, 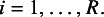 The first term in (7) represents the squared bias and the second term is the sampling variance.

#### 5.6.5 Coverage proportions

An additional way to evaluate the bias in parameter estimates and standard error estimates is to look at the coverage proportions of the 95% confidence intervals. For each replication, we checked whether the population value is in the reported range of the 95% confidence interval around the parameter estimate and calculated the percentage of replications in which the 95% CI includes the population value. For 1,000 replications, the 95% CI around a value of 0.95 is [0.936, 0.964], and we used this range of values to judge whether the coverage proportion values reflect the expected value of 0.95.

#### 5.6.6 False positive rate

For the conditions where the direct path from the independent variable X to the latent variable Y was kept at 0, we could calculate the percentage of replications where the analysis points to a result that is significantly different from 0 for that parameter. This is an estimate of the Type I error, which should be equal to the 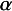 value set for the significance test. In the current study, our 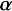 value is set at 0.05, and the 95% CI around a value of 0.05 is [0.036, 0.064], which is the range we used to assess whether the actual false positive rates correspond to 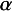 .

#### 5.6.7 Power

Power was estimated by looking at the percentage of replications that led to a significant Wald-test for a path coefficient when the population value is in fact different from zero. The null hypothesis for this test is that the population value associated with that parameter equals 0, and when the population value is indeed different from 0, the null hypothesis should be rejected. In the literature, the general advice is aiming for at least 80% power,^40^ which was the criterion we used in this study.

## 6 Results

The following sections detail the results from our simulation studies separately for each of our nine evaluation criteria, excluding the standard deviation of parameter estimates as they had a very similar pattern to RMSE results. In the following sections, we identified whether a simulation design characteristic is influential or not by looking at the plots to see if the levels of a factor show variation in results that go beyond the range of acceptable values. If all values associated with a characteristic were within the range of acceptable values, even though there were differences across the levels of the design characteristic, we did not identify it as influential. The exchangeability of effect sizes had been identified as an influential simulation design characteristic only for the method of largest effect size selection, which is why it is not included in the discussion and displays of the results going forward. Moreover, the differences in results across the different parameters, that is, 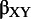 , 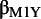 , and 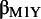 , were not systematic and are therefore not discussed in detail.^vi^ Instead, we focus only on results for the 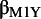 parameter moving forward. Additionally, the findings below are all concerning Stage 2 results, since ultimately the parameter estimates in the SEM model are of main interest for applied researchers. This decision was also supported by the similarity in results across the two stages. Interested readers are encouraged to look at the supplementary materials where they can find the relevant plots and explanations.

### 6.1 Convergence

All methods performed satisfactorily in terms of convergence; the highest percentage of non-convergence was lower than 0.1%. For the details of the percentages of non-convergence across the different conditions for the different methods, interested readers can refer to the Supplementary Material.

### 6.2 Relative bias in parameter estimates

Across all the conditions, the largest bias was observed for the largest effect size selection method. Choosing the effect size reporting the highest correlation coefficient amongst the multiple effect sizes for the same bivariate relation resulted in high bias reaching 150% in some conditions. Since the bias was quite extreme, the largest effect size selection approach is not included further in the results for the other evaluation criteria. The detailed plots for the separate methods encompassing all conditions and all coefficients can be found in the supplementary materials.

**Figure 3:**
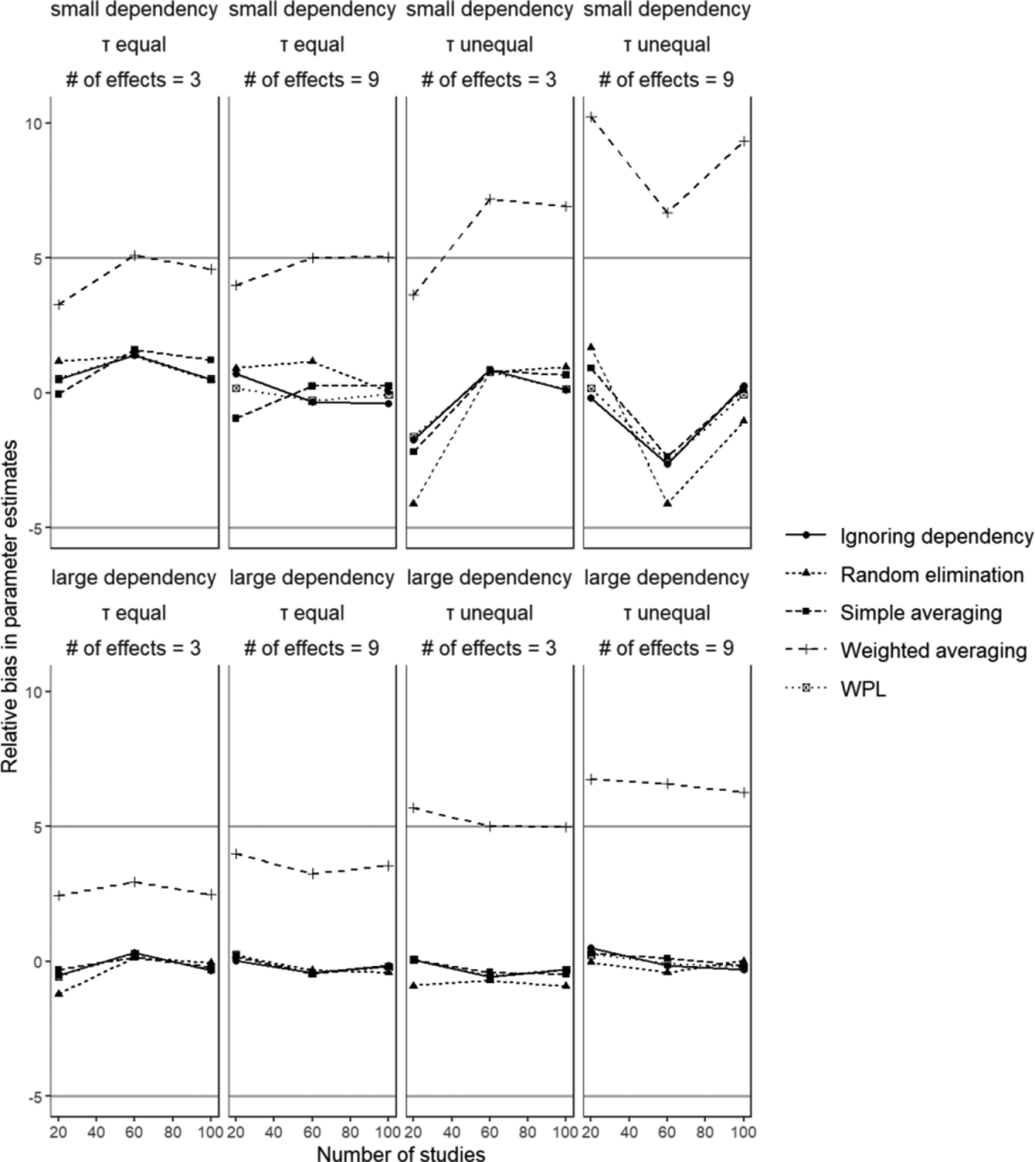
Relative bias in parameter estimation 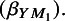 The gray lines mark range of acceptable bias (|5|%).

Figure 3 shows the percentages of relative bias for 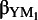 in conditions with small disparity between dependent effect sizes. Simple averaging and random elimination showed acceptable performance across all conditions with relative bias within the 5% range. The ignoring dependency and the WPL approaches also showed similar performances with both showing bias less than 2% in all conditions. Weighted averaging, on the other hand, had a worse performance with bias higher than 5% in some conditions. Looking at the plot in Figure 3, we can see that in conditions where between studies variances are unequal, the bias values for weighted averaging go higher than the range of acceptable values. The bias was especially worse for small dependency, when the between studies variances are not equal, and/or when the number of effect sizes is 9, overestimating the coefficient up to 10%. This finding makes sense; the weighting scheme used in the current study ends up giving more weight to dependent effect sizes that have higher values since the within-study sample sizes associated with them are equal. Thus, in the presence of more varied dependent effect sizes, the difference in weights becomes more pronounced, with larger effect sizes having larger weights, thus leading to a higher synthesized value. This can be easily seen in the Stage 1 results provided in the supplementary materials. Using a higher value for that correlation coefficient then leads to the overestimation of the population effect size. Manipulation of the size of dependency, number of studies, and number of effect sizes did not seem to have a consistent effect across all methods; all bias values are within the indicated range of acceptable values.

### 6.3 Relative bias in standard errors

**Figure 4:**
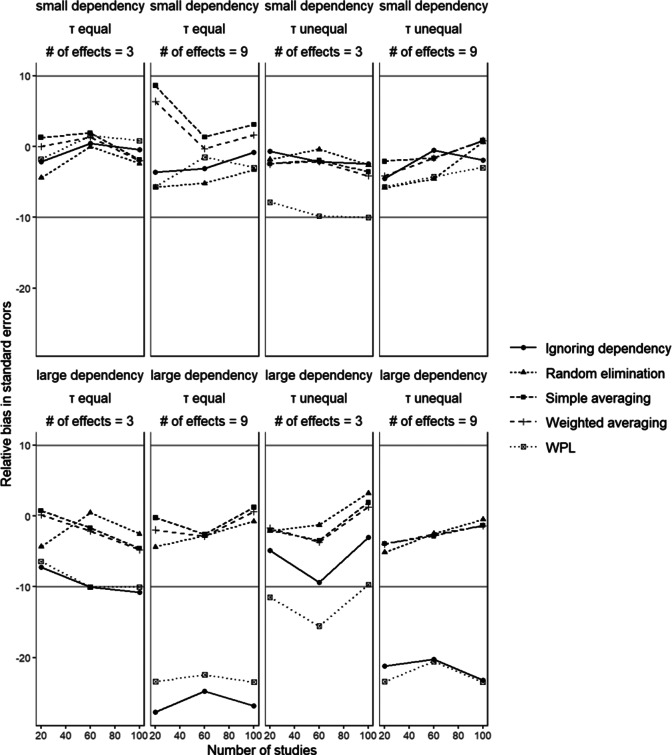
*Relative bias in standard errors* 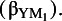 The gray lines mark the 10% bias lines to mark the range of acceptable bias.

Figure 4 shows the percentages of bias in the standard errors associated with 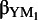 in conditions with small disparity between dependent effect sizes. Simple averaging and weighted averaging generally showed acceptable amounts of relative bias in standard errors across the different conditions.^vii^ Both methods performed worst when the number of effect sizes was 9 and when the size of dependency was small, reaching a 10% bias. With random elimination, the bias was also within the acceptable range across all conditions,^viii^ with higher bias with a greater number of effect sizes, though this trend is not as visible as in the two aggregation methods. The results were less favorable for the WPL approach and for ignoring dependency. The ignoring dependency approach showed underestimated standard errors in conditions in which dependency is large and the number of effect sizes is 9. The bias reached −30% in these conditions. As the number of effect sizes increased, the ignoring dependency approach ended up with more dependent effect sizes being treated as unique pieces of information. This then meant that there was a larger overlap in the information that gets ignored, hence a larger bias. The same logic also applies when the size of dependency increases; larger dependency means a higher correlation between the dependent effect sizes, and thus, ignoring a larger amount of dependency leads to higher bias. Large bias in standard errors could also be seen in the WPL approach, especially with larger dependency between effect sizes and more effect sizes. In these conditions, the method led to 20% negative bias. The WPL approach and the ignoring dependency method only showed acceptable performance with small dependency, and when the number of effect sizes is 3.

Comparing the methods across all the conditions, we can see that the best and most consistently performing methods are the two aggregation methods, followed closely by random elimination. In general, having unequal between-studies variances did not indicate a systematic change in bias. On the other hand, for the methods that show bias, as the number of dependent effect sizes increased the bias also increased. This is understandable given the fact that as the number of dependent effects increases, the implications of dependency on the results also become more substantial. Varying the size of dependency also had a substantial influence on the results; as we moved from small to large dependency, the performance of all six methods suffered, with most showing values higher than 5% bias. This trend was especially visible for the WPL approach and ignoring dependency, which showed the worst performance. This finding makes sense as the larger the dependency between effect sizes, the higher the bias will be if the dependency is not taken into account appropriately. We had originally expected the WPL method to be able to deal with this dependency better than the other methods, but the assumptions of the method proved to also have implications on this regard. This is of special interest, since in the realm of dealing with dependent effect sizes in the context of MASEM, the WPL approach is the suggested approach given the fact that it explicitly models the dependency. However, we showed here that even though the WPL approach performed well in terms of parameter estimation, it did not perform up to the standards in terms of bias in standard errors, which substantiates the need for better methods.

### 6.4 Absolute bias in parameter estimates

**Figure 5:**
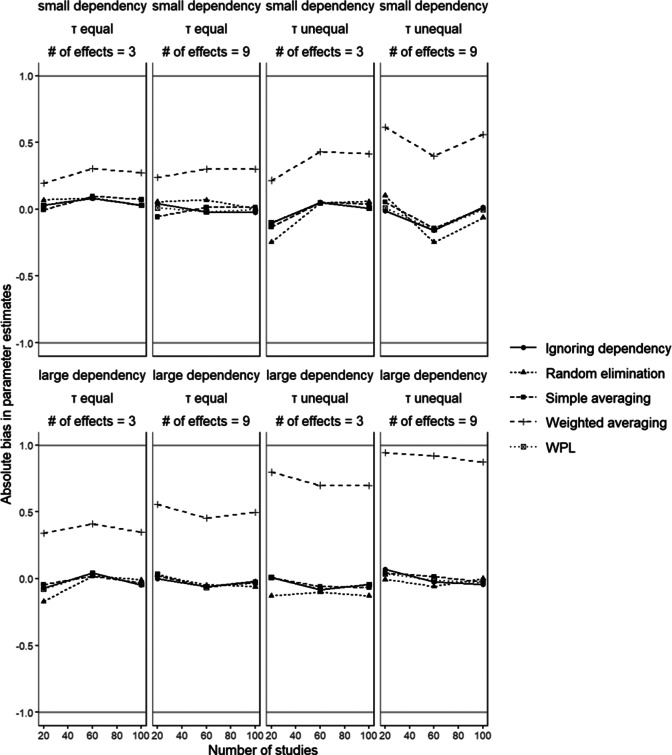
Absolute bias in parameter estimation 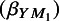 .

Figure 5 shows the absolute average bias values in parameter estimates for 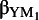 in conditions with small disparity between dependent effect sizes. We observe a repetition of the patterns as in the relative bias in parameter estimates, where weighted averaging shows the highest bias values and all other methods show comparable performance. As before, the bias in weighted averaging increases with a larger number of effect sizes, and with unequal between studies variances. We also observed larger bias in conditions with large dependency.

Manipulation of the other design factors did not seem to have a consistent effect across any method.

### 6.5 Absolute bias in standard errors

**Figure 6:**
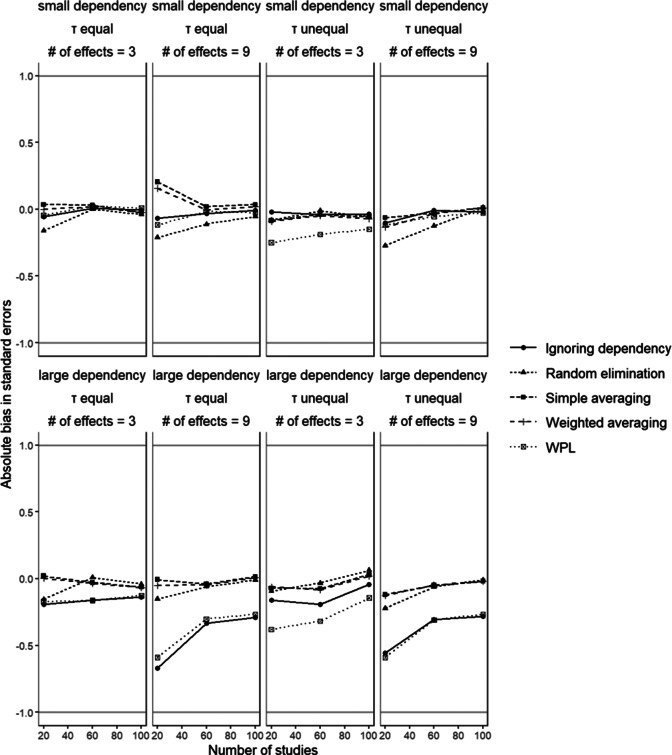
Absolute bias in standard errors 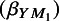 .

Figure 6 shows the percentage of absolute bias in the standard errors associated with 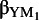 in conditions with small disparity between dependent effect sizes. We again observed similar patterns with the relative bias results; the ignoring dependency and WPL approaches showed the highest bias values. This bias was larger in conditions with large dependencies and larger number of dependent effect sizes. As the extent of the dependency increases, the implications of said dependency on the results also become more visible. The remaining three methods did not show much variation across the different conditions and had similar performance. Manipulation of sample size or between-studies variance did not seem to have a consistent effect across any method.

### 6.6 Root mean squared error

**Figure 7:**
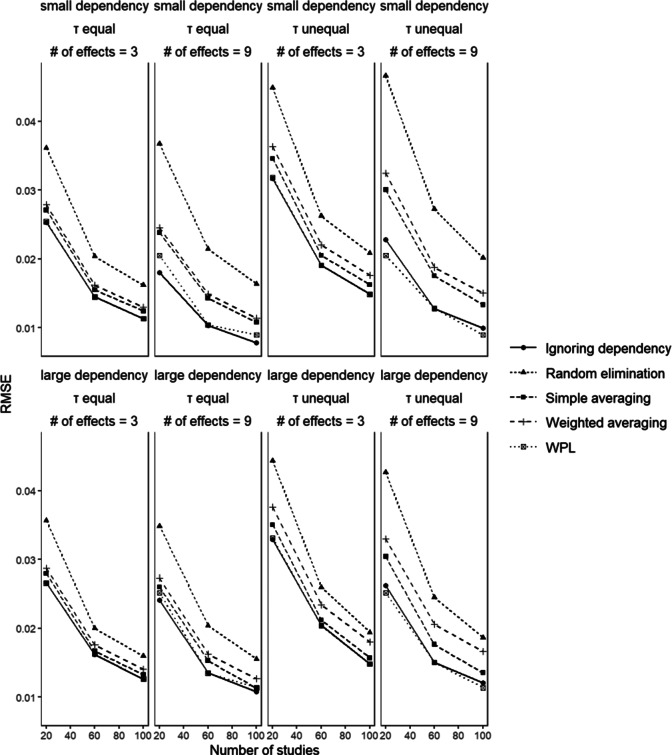
*Root mean squared error values of the parameter estimates associated with* 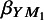 .

Figure 7 shows the root mean squared error values for 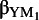 in conditions with small disparity between dependent effect sizes. It can be seen that there is not much variation in values across the different conditions and methods. Generally, in conditions with 9 dependent effects, the ignoring dependency and WPL methods show the lowest RMSE values. In the conditions with 3 dependent effects, the difference between the methods is not distinguishable. For the random elimination and the two aggregation methods, a change in the number of dependent effect sizes does not lead to change in RMSE, whereas for the two remaining methods a change in performance is more visible. Across all conditions, the random elimination strategy showed the highest RMSE values.

Across all methods, we can see that as the number of studies included in the analysis increases, the RMSE values decrease, which was expected. We also observed worse performance for all methods in conditions where the between-studies variances are unequal. In conditions with unequal between-studies variances, we observe more heterogeneity in effect sizes, which then leads to more variance in estimates. As eluded to before, the pattern of results was very similar for the standard deviation of parameter estimates, which is also used as a measure of efficiency. Given that RMSE is a measure of bias and variance combined, the similarity between the results for these evaluation criteria points us to the conclusion that the bias in parameter estimates is not large enough to have made a difference. We also observed how small the absolute bias in parameter estimates was in Figure 4, which corroborates this conclusion.

### 6.7 Percentage of false positives

**Figure 8:**
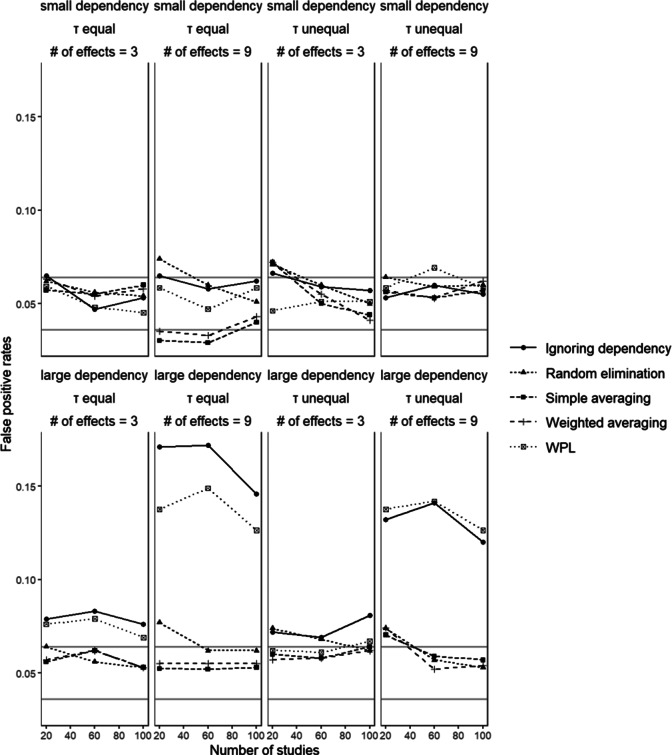
*False positive rates of the significance test for the path between X and Y.* The gray lines mark the range of acceptable values around 0.05.

Figure 8 shows the percentage of false positives for 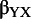 in conditions with small disparity between dependent effect sizes. Since 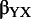 was the only parameter for which we included a true population value of 0, it is the only parameter we can then calculate the percentage of false positives for. For simple averaging, the values were mostly above 5% across the different conditions with some reaching 8%. When the number of effect sizes increased the percentage of false positives decreased. There was also a slight pattern with regard to the size of dependency; the percentages seemed to generally increase when the dependency was large, specifically when the between-studies variances were equal. Weighted averaging showed the same patterns as simple averaging with the lowest rates observed when the dependency is small, the number of effect sizes is 9 and the between-studies variances are equal. Manipulating any of these variables led to higher rates with some conditions reaching 8%. Random elimination generally showed values above 5% and the variations around this value did not follow a systematic pattern across the conditions. The lowest percentage was observed with small dependency when the number of effect sizes is 3 and the between-studies variances are equal. In the ignoring dependency approach, the percentage of false positives increased above 15% with a larger number of effects when a dependency is large. Whereas for the conditions with three effect sizes the effect of the size of dependency was dismissible, in conditions with nine effect sizes a larger dependency led to a value as high as 18%. The effect of manipulating the number of effect sizes was also visible in the WPL approach, where the percentage of false positive results increased with increasing the number of dependent effect sizes. There was also a clear effect of size of dependency, where regardless of the number of effect sizes, a large dependency led to higher percentages compared to when dependency was small, owing to the larger correlation between effect sizes.

Comparing all the methods, the ignoring dependency and WPL approaches had the highest percentages of false positives. The best-performing methods with the lowest and most consistent percentage of false positives were the two aggregation methods, with random elimination close behind. In general, as the number of effect sizes increased, so did the percentages for every method, a pattern that is most visible when the dependency is large. Higher values were observed for each method when the dependency is large as opposed to when it is small. As the extent of dependency between the effect sizes increased, the implications of said dependency on the results became more visible. These results were also in line with the results from the relative bias in standard errors since when standard errors are underestimated, Type I error rates get inflated.

**Figure 9:**
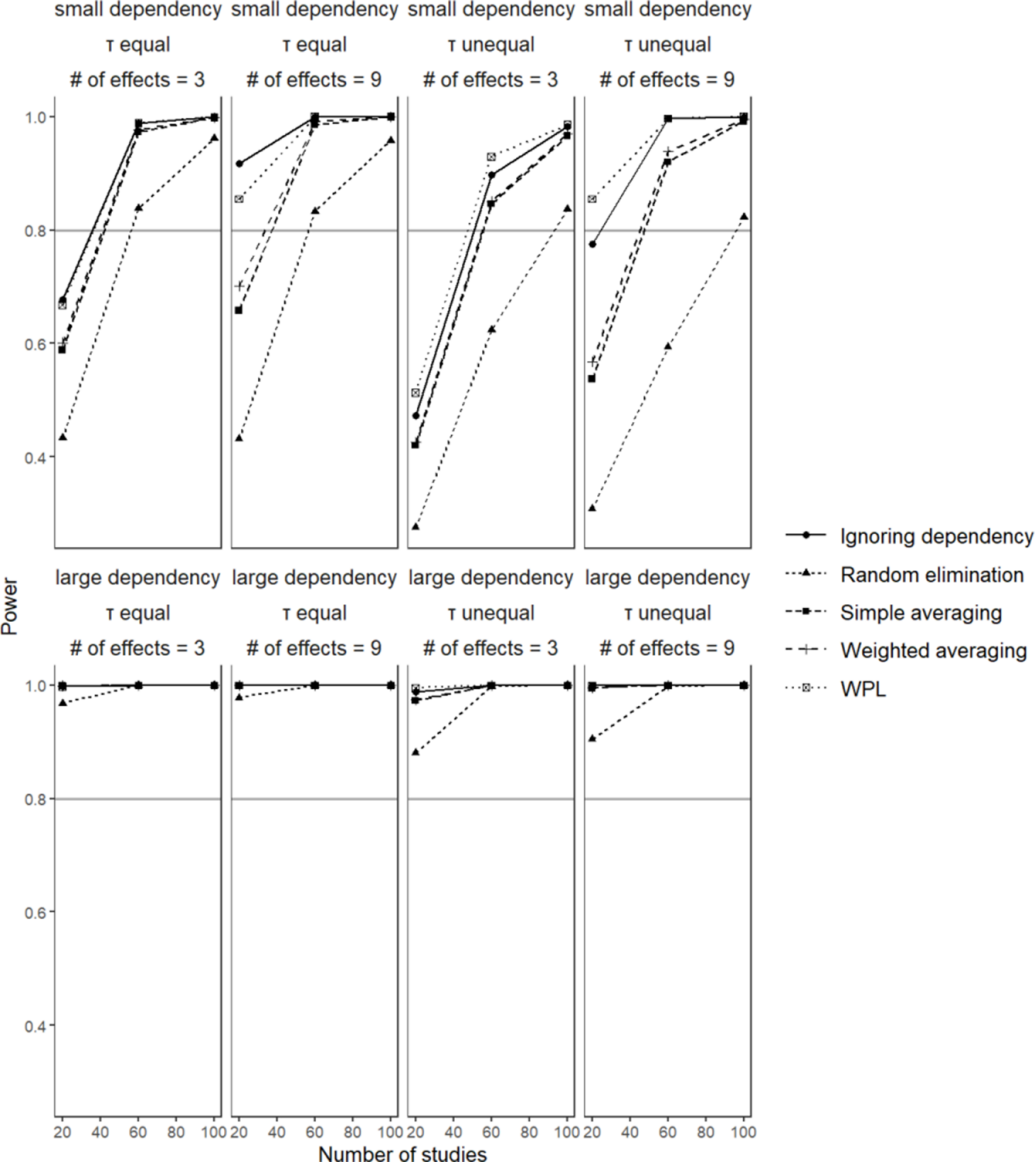
*Power of the significance test for* 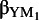 . The gray line marks the 80% line.

### 6.8 Power

Figure 9 shows the statistical power to detect a significant result for 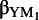 in conditions with small disparity between dependent effect sizes. A clear trend of increasing power with a higher number of studies can be observed for all methods. Both simple averaging and weighted averaging performed best when the dependency was large; as the reliability of the indicators leading to the dependent effect sizes increased, we got more relevant information to be able to calculate the population parameter estimates. In cases where dependency is small and the number of studies is lower than 60, power was below the 80% threshold. For random elimination, the poor performance was widely visible; for the conditions where dependency is small, sufficient power was only observed when the number of studies was 100. The method performed best when dependency is large, and the number of studies is large. The results for the ignoring dependency and WPL approaches were not as relevant since we had established the high false positive rates associated with these two methods. The fact that these two show the highest values for power does not mean as much since they also indicated significance when the true population value was 0.

Comparing all methods, power was highest for the ignoring dependency and the WPL approaches since these made use of all available information, but they also had the highest false positive rates, which puts the finding of high power under a different lighting. The worst performing method, on the other hand, was the random elimination method, which loses the most amount of information as it only makes use of one effect size amongst many in estimating the parameter values. In terms of the general trend across all methods, holding other variables constant, power increased with higher sample size as expected, as well as with increasing dependency. The number of effect sizes and the between-studies variance were not influential factors for any of the methods.

### 6.9 Coverage proportions

For simple averaging, weighted averaging and random elimination values of coverage proportion mostly ranged between 90% and 97% with no systematic change in the pattern across the different conditions. These methods had the highest coverage proportion when the number of effect sizes was 9, dependency was small and the between-studies variances were all equal, however, there were values below 95% in each set of conditions. The ignoring dependency approach showed a clear trend of smaller coverage proportion with the increasing number of dependent effect sizes, especially when the dependency is large, reaching values lower than 84% in some conditions. The WPL approach also showed the same pattern; in conditions where the number of effects is 9 and dependency is large, the coverage proportions were as low as 85%.

**Figure 10:**
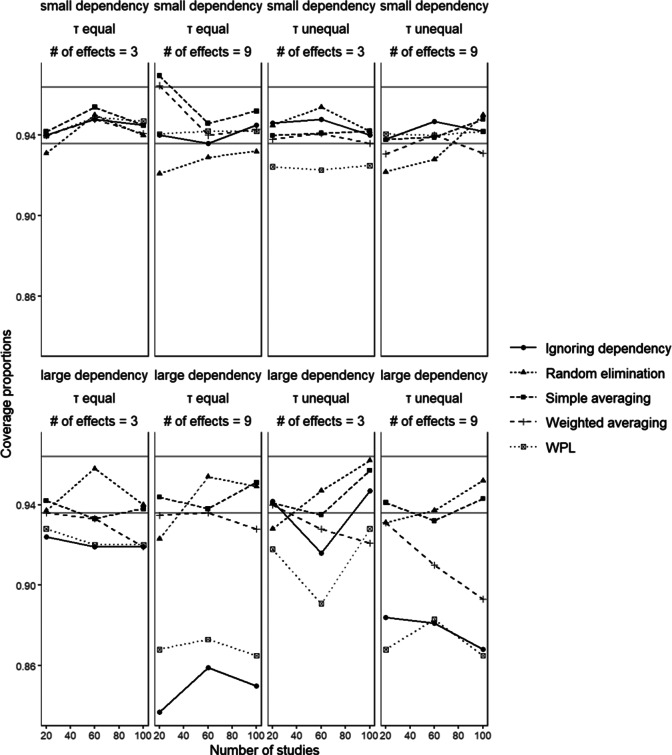
*Coverage proportions of the 95% confidence interval (* 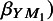 . The gray lines mark the range of acceptable values around 0.95.

Figure 10 shows coverage proportions of the 95% confidence intervals for 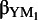 in conditions with small disparity between dependent effect sizes. Comparing all the methods, the ignoring dependency and the WPL approaches could be identified as the worst-performing methods. This was especially evident in the conditions where dependency is large and the number of effects is 9, where there was large undercoverage for both methods. The remaining three methods had comparable performance and did not differ substantially from each other across the different conditions, with the exception of the weighted averaging method showing a visibly worse performance in conditions with large dependency and unequal between-studies variances. These findings were in line with the results from the relative bias in standard errors, as standard errors being underestimated led to confidence intervals being too narrow, which resulted in smaller coverage proportions.

## 7 Summary of the results

In terms of relative bias in effect size estimates, the largest effect size selection method had the worst performance with values reaching 150% in some conditions. Among the remaining methods, weighted averaging performed the worst with a positive relative bias reaching 10%. All other methods showed acceptable performance, with some variation across the different conditions. The two aggregation methods were the best and most consistently performing methods in terms of bias in standard errors. The WPL approach and ignoring dependency showed the worst performance with large underestimation in standard errors. In terms of RMSE values, across all conditions, random elimination showed the highest values. The remaining four methods had very similar performance, with the WPL and ignoring dependency methods showing lower values with more dependent effects, since these two methods made use of all available information. The ignoring dependency and WPL approaches had the highest power, but also unacceptable false positive rates. The best-performing methods with the lowest and most consistent false positive rates were the two aggregation methods, with random elimination close behind, but the random elimination method showed the lowest power. Finally, the ignoring dependency and the WPL approaches were identified as the worst-performing methods in coverage proportions, which followed by the fact that they largely underestimated the standard errors.

In terms of Stage 1 results, as mentioned previously we generally saw the same trends as in Stage 2. Largest effect size selection also resulted in very high relative bias in correlation estimates, and the remaining methods showed acceptable values across all conditions, with the exception of weighted averaging. The overestimation of the correlation coefficient between the M1 and Y variables then led to the overestimation of the parameter estimate for the path between M1 and Y. Ignoring dependency and WPL approaches were again identified as the worst-performing methods when it came to relative bias in standard errors, with significant underestimation reaching 30%. The remaining three methods showed comparable performance. The random elimination method had again the highest 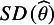 values indicating inefficiency due to the loss of information resulting in loss of precision. In terms of RMSE, the WPL and ignoring dependency approaches showed the lowest values. The two aggregation methods followed close behind, and random elimination had the highest values across all conditions.

## 8 Discussion

The most interesting finding of our study is that the WPL approach is not the best-performing method, as it leads to substantial underestimation of standard errors, which then results in higher false positive rates and lower coverage proportion rates. This is quite a crucial finding given that prior to the results from this simulation study, the overarching advice when it comes to what method to use in the presence of dependent effect sizes is the WPL method.^10^^,^ ^20^ The reason behind the bad performance of WPL may be the unrealistic assumptions regarding the random-effects. As discussed in the introduction, WPL assumes equal variances of random effects and sampling error for the different cells in a correlation matrix (see Equation (3)). This assumption apparently leads to faulty results when the different correlations in reality operate in different ways as was reflected in our simulation conditions.

Taking into account all the evaluation criteria, the best-performing method has instead been identified as simple averaging since it consistently had higher power and efficiency across the different conditions compared to random elimination. This finding is positive considering that most researchers already use simple averaging when they face dependent effect sizes, and it gives us confidence in the fact that their findings are not overly biased. However, even though simple averaging has been identified as the best-performing method here, there are still some drawbacks associated with it, mainly in terms of power and false positive rates. What we found is that with lower values of dependency, a sample size of 20 did not result in sufficient power. We also generally saw false positive percentages higher than 5%, which means that the method overestimates the significance of the results when the population value is actually 0. In terms of conducting meta-analyses, these findings bring forward important issues, since the aim would be to synthesize the available findings in an accurate way and to be able to distinguish significant findings from non-significant ones. It is also important to note that, even though it shows acceptable performance in terms of bias in parameter estimates and standard errors, simple averaging suffers from a theoretical perspective in that it fails to model the dependency between the effect sizes. By creating a new aggregate value, the method not only ignores the heterogeneity between effect sizes and limits the extent of questions we can ask especially with regard to potential moderators, but it also then treats this value as an effect size, when in reality that exact value has not necessarily been observed. With this method, there is also no way to distinguish between a study that only reports one single effect size and one that reports many.

## 9 Limitations and future research

While the findings from our study give us a lot of insight into how well the different methods perform, it is important to establish the limitations associated with our setup. In order to be able to simulate MASEM data with dependent effect sizes, we chose to create a population model with a latent Y variable of which the correlation between its indicators and other variables acted as the multiple effect sizes. Since we wanted the resulting matrices to be correlation matrices, this limited the values we could use for the factor loadings. Future studies could use other population values for factor loadings and instead generate covariance matrices to see if the results would be different. Another related point is with regards to how the chosen factor loading values translate to the correlation between the dependent effect sizes. Our chosen values of 0.7 and 0.3 lead to correlations of 0.48 and 0.09 between effect sizes, respectively. While these values are in agreement with the values used in other simulation studies (e.g., Van den Noortgate, 2013), it does not reflect the full range. It could be interesting to see how the results would differ in cases where the correlation between dependent effect sizes is higher and also when it is exactly zero. Van den Noortgate et al.^25^ showed that the standard errors associated with the ignoring dependency approach are only correctly estimated when there is no correlation between effect sizes, which we expect to also apply in the context of MASEM.

In the current simulation study, for purposes of simplicity, we opted to not include moderator variables. Yet, given the nature of meta-analysis where researchers try to understand the heterogeneity between studies and try to detect a pattern, moderator analysis is quite beneficial in general. Future studies should therefore generate datasets with moderator variables and assess the performance of the six methods investigated here. The drawbacks of the simple averaging approach will be more evident in scenarios where moderator variables are of interest since a moderator could be a characteristic of the effect sizes. For ease of computation, we also opted to have dependent effect sizes only for the outcome variable, which does not fully reflect a real-life scenario as the multiple studies included in the meta-analysis rarely measure exactly the same independent and mediator variables multiple times. The same data generation strategy can be used to introduce more dependent effect sizes for the other variables involved to see if the existence of dependent effect sizes across all the bivariate relationships would have an effect on the results. Similarly, the prevalence of dependent effect sizes was kept constant at 70% across all the conditions. While this closely reflected values reported in the literature, it would be interesting to see if and how manipulating this factor would change the results. In the current study, to reflect the real-life scenario of missing data we also enlisted a random deletion procedure where we deleted 15% of the correlations reported excluding the dependent effect sizes. This was a non-manipulated factor, and it would be interesting to see how much the different methods would suffer as the missingness in the data increased.

Our choice of data generation mechanism can also be seen as a limitation since we assume that different studies make use of the same indicators, when in reality it could also be that these indicators are only a random selection from a population of indicators. This mechanism is obviously harder to model, which is why we chose to make that assumption to make things computationally more straightforward. Future studies could employ different data generation mechanisms to reflect this scenario. In our population model, the only path coefficient we had changed the values of was the path between the X and Y variables, which was mainly done to be able to calculate false positive rates. Future studies could also investigate the effects of changing the values for the path coefficients concerning the mediator variables to assess whether the size of the relationship between variables then attenuates or inflates the effect of dependent effect sizes. Similarly, we also opted to keep the population model the same across the conditions of the simulation study, as the main focus here is to first see the influence of choosing different methods for handling dependency on the results of the analyses. Future research could focus on whether generating data using other population models with different number of independent, dependent, and mediator variables than the model used in the paper would have a different influence on the results. In hindsight, our manipulation of the between-studies variances was also not ideal, given that in the condition where they are unequal, they are also larger on average than in the condition where the between-studies variances for the dependent effect sizes are equal. Thus, the difference in performance due to the manipulation of this factor is not only because of the between-studies variances being unequal but also being larger. Moving forward it will be smart to include a level to this factor where the between-studies variances are all equal and large, or to modify the inequality condition to have the same average value as the equal condition.

Moving forward, what is perhaps the most crucial point to address is what steps can be taken to make the methods discussed in this paper better. As previously mentioned, even though simple averaging has performed the best in the setup of the current simulation study, it suffers from a major setback of not actually addressing and modeling the dependency in effect sizes. On the other hand, the only method which does address the dependency results in underestimated standard errors. This brings forth the possibility of incorporating other methods that are able to give unbiased standard errors in the presence of dependency, such as robust variance estimation (RVE). Researchers have shown RVE to be a valuable technique in the presence of dependent effects.^14^^,^ ^41^ In their paper, Fernández-Castilla et al.^42^ show that by applying an RV correction, the bias in standard errors can be cut down more than halfway. Amongst the models they assess the performance of is a three-level model with one random study effect, which resembles the WPL method very closely. They also provide a three-level model with separate random study effects, which would be the natural extension of WPL as well. Future research could assess how to incorporate this in the context of MASEM, to hopefully come up with a better solution to handle dependent effect sizes.

## 10 Conclusion

The aim of this paper was to compare the various strategies researchers currently use when they are faced with multiple dependent effect sizes in the context of MASEM and to substantiate the need for better-performing methods. The WPL method was not identified as the best-performing method because it showed substantial negative bias in standard errors, and hence inflated false positive rates and small coverage proportions. While the simple averaging method had been identified as the best-performing method across the conditions of the simulation study, it still suffers from both theoretical and practical drawbacks. Therefore, we call for the development of better methods for handling dependent effect sizes in MASEM.

## Supporting information

Bilici et al. supplementary material Bilici et al. supplementary material

## Data Availability

All simulated data and code used in the paper are available here: https://osf.io/nvswq/.

## Notes

i. When we write “dependent effect sizes” we refer to multiple coefficients for the same bivariate relationship. Another type of dependent effect sizes is the dependence between effect sizes for different bivariate relationships within the same study (the elements in a study’s correlation matrix). That dependency, leading to sampling covariance, is taken into account by applying multivariate meta-analysis at Stage 1.

ii. In our simulation study (discussed later) we will evaluate the realistic scenario of not having access to the correlations between dependent effect sizes, and not being able to adjust the sampling variance accordingly. So, we use the sampling variance of the average correlation as if it was a single observed correlation coefficient. This is also why a choice between using a fixed versus random-effects model in creating a weighted average of dependent effect sizes would not have an effect on the results since we are only making use of the point estimates and not the standard errors of the estimates.

iii.
https://osf.io/nvswq/.

iv. Interested readers can find the R script detailing this calculation on the project OSF page.

v. In our setup, this distribution did not lead to any 0 values for sample size. The values obtained from this distribution are expected to center around a value of 94.16 with a standard deviation of 28.896.

vi. We conducted one-way ANOVA for each method and each evaluation criteria using the parameter as the predictor. The only method for which there were significant differences in the results depending on which parameter is being used was the weighted averaging method, where the direct path from the X variable to the Y variable (  ) showed higher relative bias in parameter estimates than the paths going from the two mediator variables. This result makes sense, since in the calculation of the relative bias, the denominator value for the (  ) parameter is smaller than for the  and  parameters.

vii. For simple and weighted averaging, bias exceeds 10% only for the path between X and Y in the condition where dependency was small, number of effect sizes is 9, the dependent effect sizes are exchangeable, the sample size is 20, and the between-studies variances are all equal.

viii. For random elimination, bias exceeds −10% only for the path between M2 and Y in the condition where sample size is 20, number of effect sizes is 9, between-studies variances are not equal, size of dependency is large, and there is small disparity between dependent effect sizes.

## Supplementary material

To view supplementary material for this article, please visit http://doi.org/10.1017/rsm.2024.10.

## Appendix

**Table A1:** Dependent effect sizes in MASEM

Study	PC_S	PC_BC	PC_CD	S_BC	S_CD	BC_CD
1	0.256	0.581	0.490	0.320	0.459	0.322
	0.655		0.258			0.286
2	0.540	0.136	0.227	0.600	0.552	0.185
	0.310	0.216		0.365	0.490	
				0.198	0.501	

PC, parental crime; S, support; BC, behavioral control; CD, children delinquency.

**Table A2:** Example of a between-studies variance–covariance matrix for the three-indicator setup

	M1-M2	M1-X	M1-I1	M1-I2	M1-I3	M2-X	M2-I1	M2-I2	M2-I3	X-I1	X-I2	X-I3	I1-I2	I1-I3	I2-I3
**M1-M2**	.01														
**M1-X**		.01													
**M1-I1**			.03												
**M1-I2**				.01											
**M1-I3**					.03										
**M2-X**						.01									
**M2-I1**							.01								
**M2-I2**								.03							
**M2-I3**									.03						
**X-I1**										.03					
**X-I2**											.01				
**X-I3**												.01			
**I1-I2**													.01		
**I1-I3**														.01	
**I2-I3**															.01
