# Supplementary material for: Six ways to handle dependent effect sizes in meta-analytic structural equation modeling: Is there a gold standard?
Source: Res Synth Methods. 2025 Mar 13;16(1):60–86. doi: 10.1017/rsm.2024.10 (PMC12621510; doi:10.1017/rsm.2024.10)
Supplement: Bilici et al. supplementary material [file S1759287924000103sup001.docx]

**Supplementary materials**

***Standard deviation of parameter estimates***

Figure 1 shows the standard deviations of the parameter estimates associated with $\beta_{\mathrm{YM}_{1}}$in conditions with small disparity between dependent effect sizes. For all methods, we saw a clear trend of higher efficiency with an increasing number of studies. Results from conditions with unequal between-studies variances generally had lower efficiency, but the trend was not very apparent. The other manipulated factors in the study did not have a visible effect on the results.

Comparing the results across the different methods, we saw that the methods making use of all the available information, i.e. ignoring dependency and the WPL approach, had the lowest values as expected. Random elimination, which only makes use of one effect size amongst many, had the highest values as the loss of information results in loss of precision, and was thus identified as the least efficient method. The two aggregation methods settled somewhere in between, as they do make use of all available information initially but use only an aggregate value in the end.

**
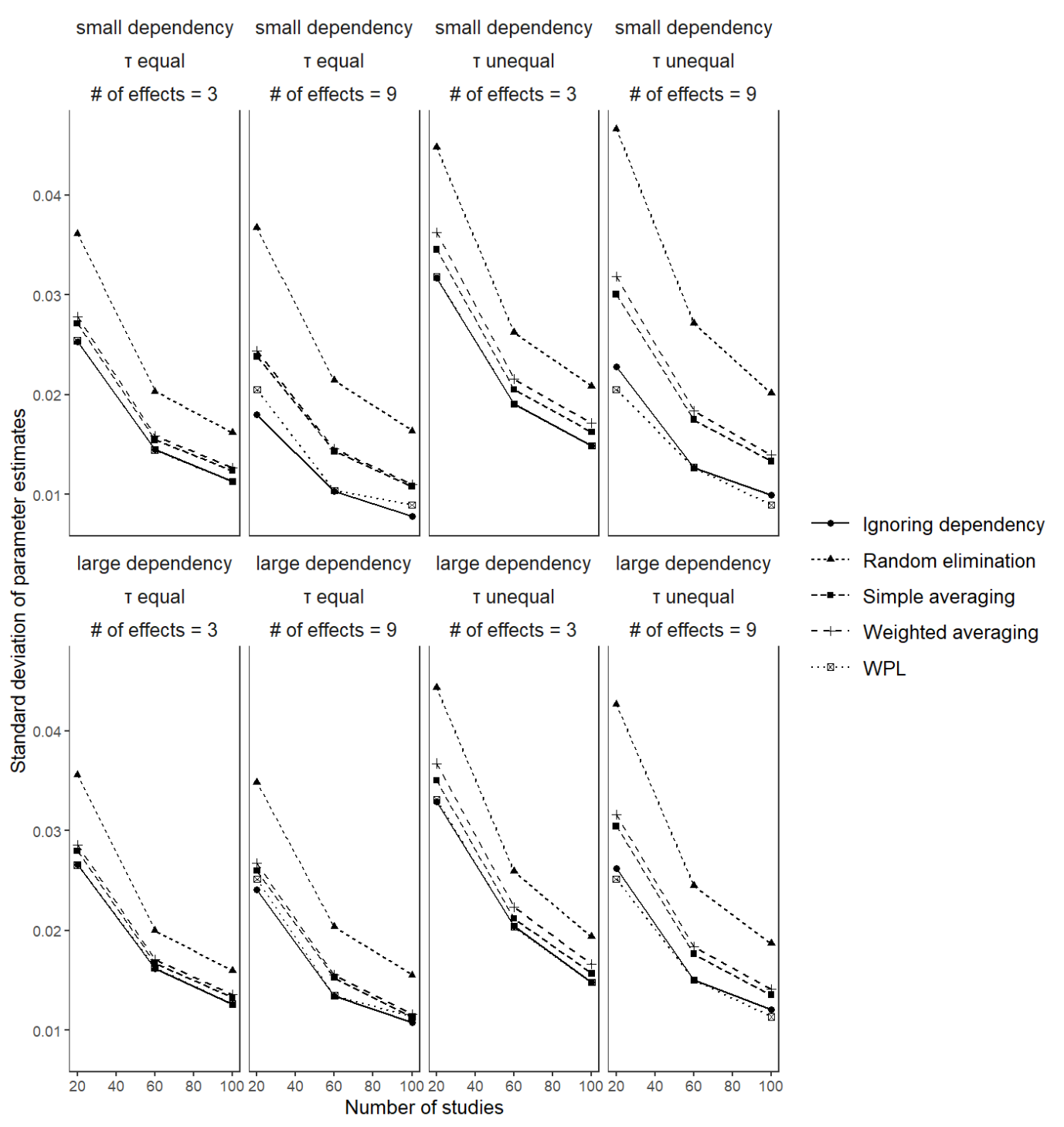
**

**Figure 1.** Standard deviations of the parameter estimates associated with $\beta_{\mathrm{YM}_{1}}$.

**Stage I results**

In the paper, all results were concerning the Stage 2 part of the TSSEM analysis, where the structural equation model is fit on the pooled correlation matrix. For each of the different methods employed in the paper, the analysis is thus the exact same in Stage 2. Therefore, any and all differences between the methods is actually due to differences in the pooled correlation matrix they estimate in Stage 1 of TSSEM. In the following sections, we take a closer look at the Stage 1 results, and assess the findings based on the following evaluation criteria: relative bias in correlation estimates, relative bias in standard errors, standard deviation of correlation estimates, and root mean squared error. Similar to the plots and discussion concerning Stage 2 results, we only looked at the estimates for bivariate correlations concerning the Y variable, which had the dependent effects present. The differences in results across these three correlations, i.e. $r_{XY}, r_{M1Y}, r_{M2Y}$, were not systematic, thus, we only focused on the $r_{M1Y}$ correlation estimate moving forward. Interested readers are encouraged to go the OSF project page to find additional plots.

***Relative bias in correlation estimates***

Across all the conditions included in the simulation study, the largest bias was observed for the largest effect size selection method. Choosing the effect size reporting the highest correlation coefficient amongst the multiple effect sizes for the same bivariate relation resulted in high bias over 150% in some conditions, which was expected. Given this quite extreme bias, the largest effect size selection approach is not included further in the results for the other evaluation criteria. The detailed plots for the separate methods encompassing all conditions and all coefficients can be found in the OSF page.

Figure 2 shows the relative bias in estimating the correlation coefficient between variables Y and M1 in conditions with small disparity between dependent effect sizes.

Simple averaging, random elimination, ignoring dependency and the WPL approach all showed acceptable performance across all conditions with relative bias within the 5% range. Weighted averaging, on the other hand, had a worse performance with bias higher than 5% in some conditions. Looking at the plot, we can see that in conditions where between studies variances are unequal the bias values for the weighted averaging approach go higher than the range of acceptable values. The bias was especially worse for small dependency, when the between studies variances are not equal, and/or when the number of effect sizes is 9. Manipulation of the number of studies, number of effect sizes, and the size of dependency did not seem to have a consistent effect across all methods; all bias values are within the indicated range of acceptable values.


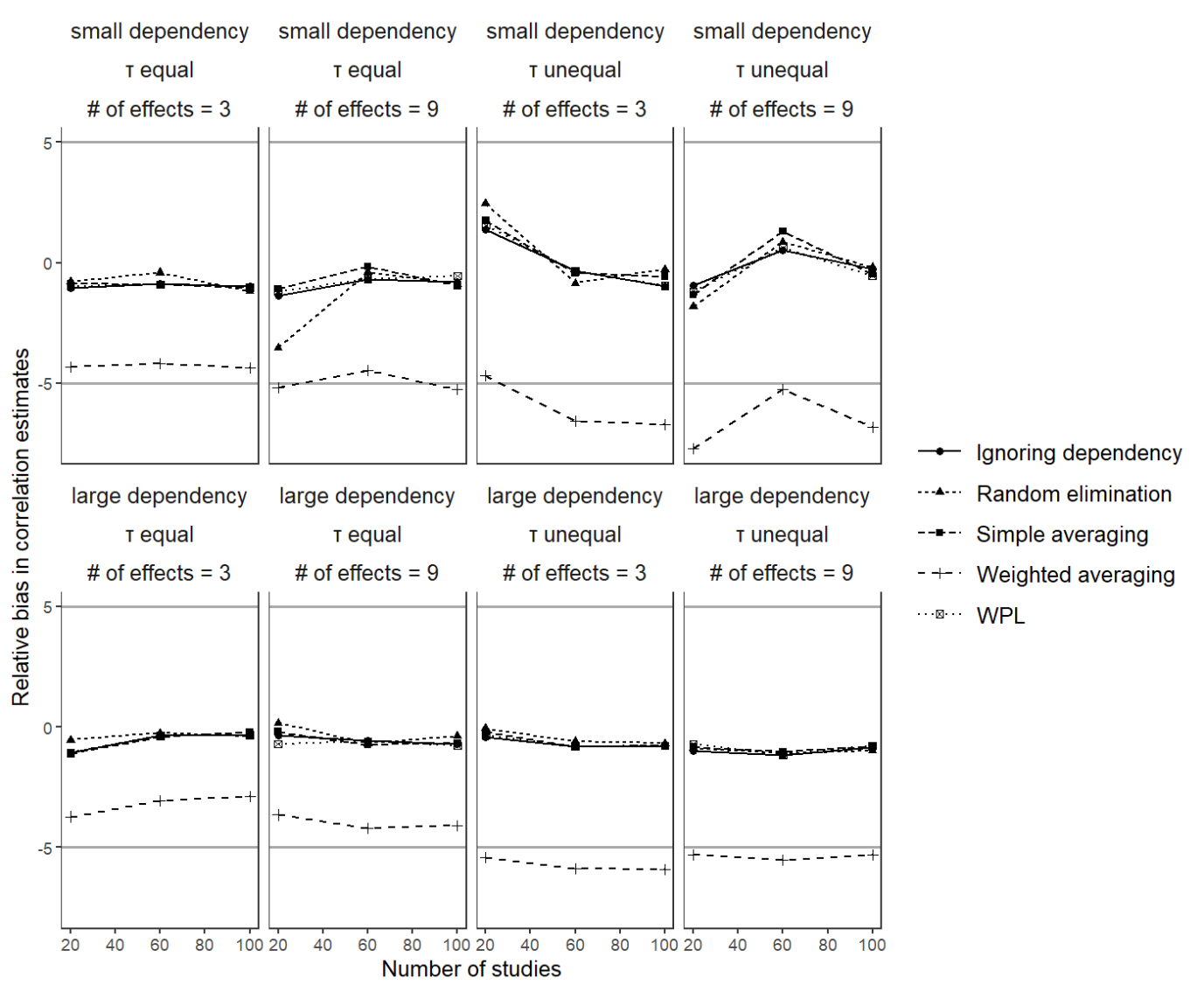


**Figure 2.** *Relative bias in correlation estimates (*$r_{M1Y}$*).* The gray lines mark range of acceptable bias (|5|%).

***Relative bias in standard errors***

Figure 3 shows the percentages of bias in the standard errors associated with estimating the correlation between variables M1 and Y in conditions with small disparity between dependent effect sizes. Simple averaging, weighted averaging and random elimination generally show acceptable values across all conditions. The two averaging methods go beyond the range of acceptable bias only in the condition when there is small dependency between effect sizes, the between-studies variances are equal, and the number of indicators equal 9. Random elimination shows deviations from the range of acceptable bias in conditions where between-studies variances are unequal and when sample size is 20. The ignoring dependency and WPL approaches show unacceptable bias in more cases. The ignoring dependency approach performs the worst when the dependency between effect sizes is large and when number of dependent effect sizes is 9, sometimes reaching over negative 30% bias. These findings make sense, since as the number of dependent effect sizes increases, the overlap in information also increases. By ignoring this overlap, the method ends up underestimating the uncertainty in estimates. Similarly, when a larger amount of dependency between effects is ignored, the amount of information is overestimated which also leads to underestimated standard errors. The WPL method generally performs better than the ignoring dependency approach, but still reaches negative 20% bias in some conditions, mainly when dependency is large and when there are 9 dependent effects.

When comparing methods across all the conditions, we can identify the two aggregating methods as the best and most consistently performing methods, followed by the random elimination method. In terms of general patterns observed for all methods, we can see that as the number of dependent effect sizes increases, the performance of the methods worsens. The same applies for when the amount of dependency between effect sizes increases. As the extent of the dependency grows larger, either through correlations between the effects or by the number of effects, the implications of said dependency on the results become more substantial. Number of studies included in the analysis, or the between-studies variance did not seem to indicate a systematic change in bias.


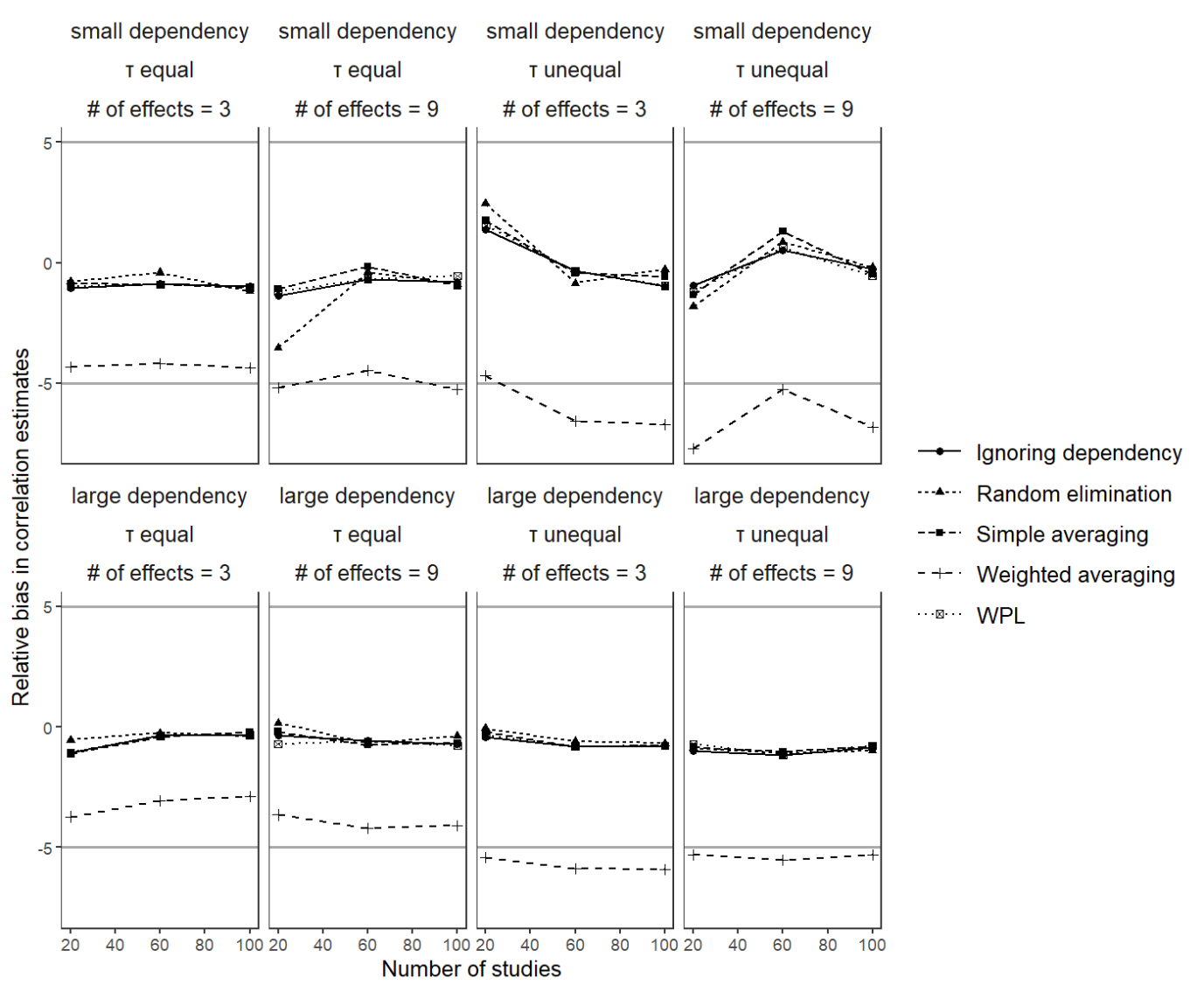


**Figure 3.** *Relative bias in standard errors (*$r_{M1Y}$*).* The gray lines mark range of acceptable bias (|5|%).

***Standard deviation of correlation estimates***

Figure 4 shows the standard deviations of the estimates of the correlation coefficient between variables M1 and Y in conditions with small disparity between dependent effect sizes. We can see a very apparent and expected pattern in terms of the number of studies included in the analysis; namely a higher efficiency with increasing number of studies. For all methods, we also observed higher efficiency when the between-studies variance was equal. Manipulating the number of dependent effects, or the amount of dependency between the effects did not have visible effects on the results.

Comparing the results across the different methods, random elimination consistently showed the worst performance, which was to be expected given that it only made use of a portion of the available information resulting in loss of precision. On the other hand, the two methods using all available information, namely the WPL approach and the ignoring dependency, had the lowest values. The two aggregation methods were somewhere in between; these methods do make use of all available information in the beginning, but end up using only the aggregate values in the actual analysis.

**
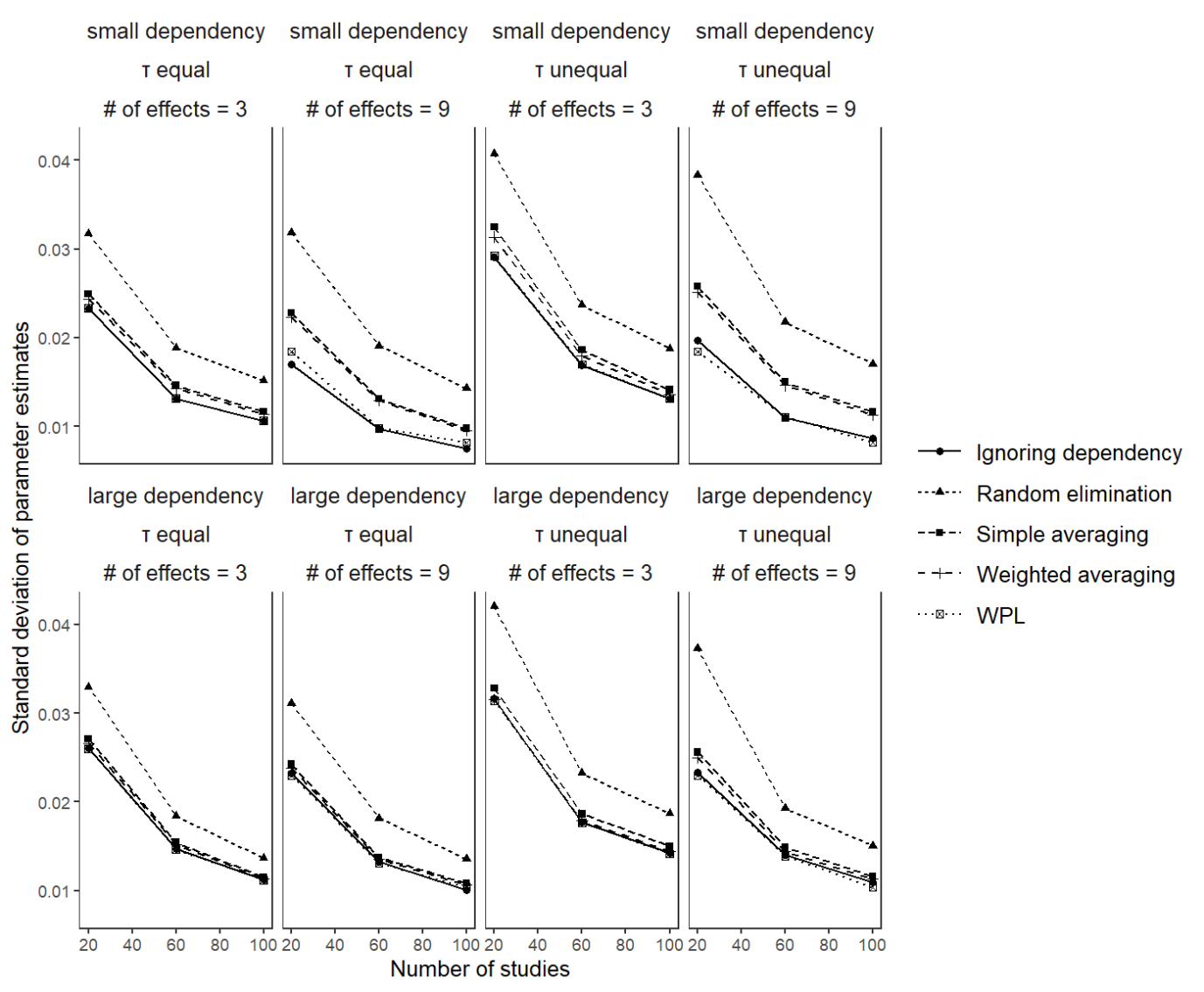
**

**Figure 4.** *Standard deviation of the correlation estimates (*$r_{M1Y}$*).*

***Root mean square error***

Figure 5 shows the root mean square error (RMSE) values associated with the estimates of the correlation between variables M1 and Y in conditions with small disparity between dependent effect sizes. We saw a clear and expected pattern of lower RMSE values with increasing sample size, given the increase in amount of available information. We also observed an increase in RMSE values with unequal between-studies variances. The other manipulated factors did not present a visible effect for all methods.

Across all conditions, the random elimination approach showed the highest values. In conditions where number of dependent effects is 3, the distinction between the other four methods was not very clear. In conditions where there were 9 dependent effects, the ignoring dependency and WPL approaches showed the lowest RMSE values, but the differences were not large.

**
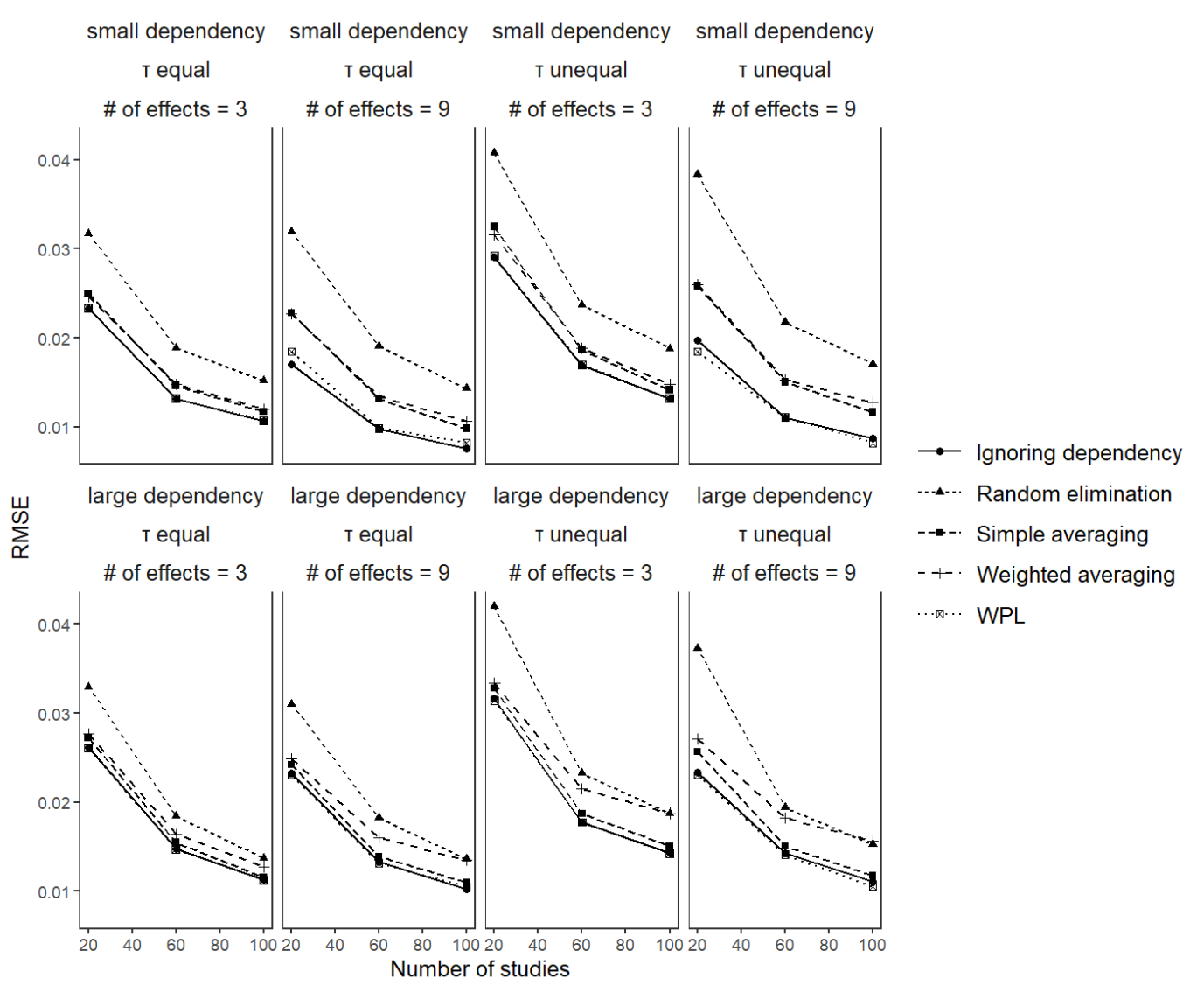
**

**Figure 5.** Root mean square error values associated with the estimation of $r_{M1Y}$.
